# A rare SNP in pre-miR-34a is associated with increased levels of miR-34a in pancreatic beta cells

**DOI:** 10.1007/s00592-013-0499-1

**Published:** 2013-07-05

**Authors:** Jonathan M. Locke, Hana Lango Allen, Lorna W. Harries

**Affiliations:** Institute of Biomedical and Clinical Science, University of Exeter Medical School, Barrack Road, Exeter, EX2 5DW UK

**Keywords:** miRNA, Single nucleotide polymorphism, Beta cell

## Abstract

Changes in the levels of specific microRNAs (miRNAs) can reduce glucose-stimulated insulin secretion and increase beta-cell apoptosis, two causes of islet dysfunction and progression to type 2 diabetes. Studies have shown that single nucleotide polymorphisms (SNPs) within miRNA genes can affect their expression. We sought to determine whether miRNAs, with a known role in beta-cell function, possess SNPs within the pre-miRNA structure which can affect their expression. Using published literature and dbSNP, we aimed to identify miRNAs with a role in beta-cell function that also possess SNPs within the region encoding its pre-miRNA. Following transfection of plasmids, encoding the pre-miRNA and each allele of the SNP, miRNA expression was measured. Two rare SNPs located within the pre-miRNA structure of two miRNA genes important to beta-cell function (miR-34a and miR-96) were identified. Transfection of INS-1 and MIN6 cells with plasmids encoding pre-miR-34a and the minor allele of rs72631823 resulted in significantly (*p* < 0.05) higher miR-34a expression, compared to cells transfected with plasmids encoding the corresponding major allele. Similarly, higher levels were also observed upon transfection of HeLa cells. Transfection of MIN6 cells with plasmids encoding pre-miR-96 and each allele of rs41274239 resulted in no significant differences in miR-96 expression. A rare SNP in pre-miR-34a is associated with increased levels of mature miR-34a. Given that small changes in miR-34a levels have been shown to cause increased levels of beta-cell apoptosis this finding may be of interest to studies looking at determining the effect of rare variants on type 2 diabetes susceptibility.

## Introduction

Recent genome-wide association studies (GWAS) have identified common (minor allele frequency (MAF) >5 %) single nucleotide polymorphisms (SNPs) associated with risk of type 2 diabetes [1, 2]. Most of these SNPs seem to exert their effect by affecting beta-cell function, rather than insulin action [3]. However, despite some success, these findings only explain ~10 % of the heritability of type 2 diabetes [1]. It has been widely postulated that rare genetic variation (MAF <1 %) could explain some of the “missing heritability” [4, 5].

MicroRNAs (miRNAs) are single-stranded, non-protein coding RNAs ~21–23 nucleotides in length that regulate gene expression by binding to mRNAs, via complementary base pairing, resulting in mRNA decay and/or translational repression. Recent studies are revealing important roles for specific miRNAs in regulating beta-cell functions, particularly glucose-stimulated insulin secretion and apoptosis (reviewed in [6]). In rodent models, during the progression to diabetes, expression of specific miRNAs has been shown to change, and manipulating the levels of these miRNAs by silencing or mimicry experiments has revealed a causal role in beta-cell dysfunction [7].

Given the need to tightly control the levels of specific miRNAs for correct beta-cell function, we sought to determine whether SNPs present within the precursor-miRNA (pre-miRNA) structure might affect their expression. Previous studies have shown that sequence variation within a pre-miRNA can affect levels of mature miRNA [8, 9]. The density of SNPs within a pre-miRNA has been shown to be lower than the SNP density in the flanking regions [10], and as a result of this constraint SNPs that are present would seem to have arisen fairly recently, meaning that the MAF is low, population-specific and not captured well by GWAS [11, 12]. Any significant associations found in this study, between SNPs with a low MAF and miRNA expression, may be of interest to future studies looking at the effect of rare genetic variation on risk of developing type 2 diabetes.

## Materials and methods

Plasmids (pCMV–MIR series) encoding human pre-miR-34a and pre-miR-96, and their flanking regions (at least 284 bp on either side), were purchased from Origene (Rockville, MD, USA). Site-directed mutagenesis was conducted using the QuikChange II XL Site-Directed Mutagenesis Kit (Agilent Technologies, Santa Clara, CA, USA). The correct sequence for each construct was confirmed by Sanger sequencing.

For all cell lines, transient transfections were performed using Nucleofector technology (Lonza, Basel, Switzerland) at a density of 1 × 10^6^ cells/transfection. After incubating for 16–24 h, cells were washed in PBS three times, and then total RNA, including miRNA, extracted using the miRVANA miRNA isolation kit (Life Technologies, Carlsbad, CA, USA).

For miRNA expression analysis, RNA was reverse transcribed using the miRNA reverse transcription kit (Life Technologies) and miRNA-specific RT primers. Subsequently, miRNA expression levels were determined using miRNA-specific TaqMan assays on the ABI Prism 7900HT real-time PCR platform (Life Technologies). The GeNorm algorithm [13] in RealTime StatMiner software (Integromics, Madrid, Spain) was used to identify the most stable housekeeping genes and thus to normalise miRNA expression levels. The following sets of genes were selected as housekeeping genes: *U6*, *4.5S* and *U87* (INS-1); *U6*, *RNU44* and *RNU6B* (HeLa); *U6*, *snoRNA412* and *snoRNA234* (MIN6). For green fluorescent protein (GFP) expression analysis, RNA was first DNase-treated using the Turbo DNA-free kit (Life Technologies), before being reverse transcribed using Superscript III reverse transcriptase (Life Technologies) and random hexamer primers. All qPCR reactions were run on the ABI Prism 7900HT real-time PCR platform (Life Technologies). A custom TaqMan gene expression assay to measure GFP mRNA was designed (Life Technologies) and its ability to robustly determine expression levels validated by standard curve. GFP mRNA levels were normalised using the GeNorm algorithm [13] in RealTime StatMiner software (Integromics, Madrid, Spain) with the following sets of genes selected as housekeeping genes: *18S*, *ACTB* and *GUSB* (HeLa); *Gusb*, *B2m* and *Actb* (INS-1); *B2m, Hmbs* and *Polr2a* (MIN6). Expression of miR-96 and miR-34a was made relative to the expression of the respective miRNA in cells transfected with the major allele of each SNP. To normalise for differences in transfection efficiency, miRNA expression was subsequently normalised to the expression of *GFP* mRNA in that respective sample. Statistical analysis was determined using Student’s *t-*test with unequal variances assumed.

## Results

In dbSNP version 135, we identified three SNPs within the pre-miRNA sequence of miRNAs important to beta-cell function (Table 1). All SNPs had to have been found in at least one HapMap individual to be included. For a miRNA to be included in our study, we required there to be evidence in the literature for an effect of over-expression or silencing of that miRNA on beta-cell apoptosis or glucose-stimulated insulin secretion. Three SNPs were identified (rs2910164, MAF = 0.38; rs72631823, MAF = 0.001; rs41274239, MAF = 0.002), two of which are located within the terminal loop region of the pre-miRNA and one within the miR* seed sequence. We chose not to study the effects of rs2910164 on miR-146a expression as this is a common SNP, and the effect on miR-146a expression has been previously extensively studied [14–16].

**Table 1:** miRNAs with a proven role in regulating glucose-stimulated insulin secretion and/or beta-cell apoptosis

	Pre-miRNA SNP?	Location of SNP	References
miR-375	No	N/A	[28]
miR-34a	Yes—rs72631823	Terminal loop	[20]
miR-146a	Yes—rs2910164	miR* seed	[7]
miR-21	No	N/A	[7]
miR-96	Yes—rs41274239	Terminal loop	[29]
miR-410	No	N/A	[30]
miR-200a	No	N/A	[30]
miR-130a	No	N/A	[30]
miR-9	No	N/A	[31]
miR-124	No	N/A	[29]

**Fig. 1:**
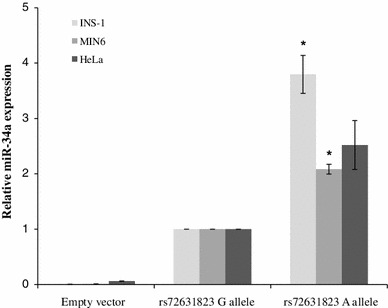
miRNA expression in cells transfected with plasmids encoding pre-miR-34a and each allele of rs72631823. For each cell line, expression is relative to cells transfected with the pre-miRNA encoding the major allele of rs72631823. Data are presented as mean ± SEM and the results of three independent transfections. Statistical analysis performed using Student’s *t-*test with unequal variances assumed. *p < 0.05

**Fig. 2:**
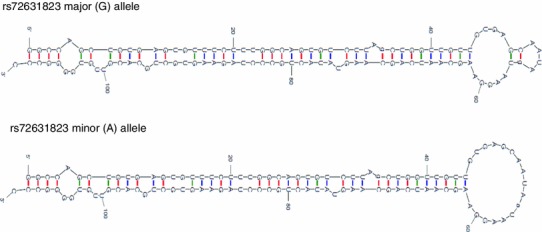
Secondary structure of pre-miR-34a for each allele of rs72631823, as predicted by Mfold

In MIN6 cells, transfection of plasmids encoding miR-34a and the A (minor) allele of rs72631823 resulted in 2.1-fold higher expression of miR-34a compared to cells transfected with plasmids encoding miR-34a and the G (major) allele of rs72631823 (*p* = 0.01) (Fig. 1). A similarly increased level of miR-34a expression from plasmids bearing the minor (A) allele of rs72631823 was seen in INS-1 cells (3.8-fold, *p* = 0.02) and HeLa cells (2.5-fold, *p* = 0.08) (Fig. 1). Using the Mfold algorithm [17] and web server (http://mfold.rna.albany.edu/), RNA secondary structure predictions support these findings. The terminal loop of the pre-miRNA bearing the A allele of rs72631823 is in a more relaxed, open form than the G allele (Fig. 2). This has been shown to be associated with more efficient Drosha and Dicer processing and consequently higher levels of mature miRNA [18]. In MIN6 cells, transfection of plasmids encoding miR-96 and each allele of rs41274239 resulted in no significant differences in miR-96 expression (data not shown). This was despite a ~eightfold increase in miR-96 expression being measured in cells transfected with plasmids encoding miR-96, and the T (major) allele of rs41274239 compared to cells transfected with empty vector. This finding is consistent with a previous study that did not identify any significant differences in miR-96 expression upon transfection of HeLa cells with miR-96 expression plasmids encoding each allele of rs41274239 [19].

## Discussion

Two studies have shown that precise control of miR-34a expression in the beta cell is needed to maintain correct beta-cell function. Experiments demonstrated that palmitate and proinflammatory cytokine-induced beta-cell apoptosis result in a threefold to fourfold increase in miR-34a expression and that specifically inhibiting miR-34a activity significantly reduces the stimulus-induced apoptosis [7, 20]. One may conclude from these experiments that miR-34a plays a causal role in beta-cell apoptosis and that small (within one order of magnitude) changes in expression, such as those observed in this study and attributable to allelic effects of rs72631823, may have significant consequences.

Our finding of increased miR-34a expression in all three cell lines transfected with the minor versus major allele of rs72631823 suggests that this may not be a tissue-specific phenomenon. Indeed, small deviations in miR-34a expression may have functional consequences in other tissues that play a role in diabetes pathogenesis. Numerous studies have found elevated miR-34a expression in fatty livers of dietary-induced and genetic mouse models of obesity [21–23]. Furthermore, higher miR-34a levels are seen in livers of human individuals with nonalcoholic fatty liver disease [21, 24]. A causal role for miR-34a in metabolic dysregulation within the liver, through regulation of the hepatic response to FGF19, has also been reported [25]. This suggests that increased hepatic miR-34a expression, which may be found in carriers of the rare allele of rs72631823, could promote a more insulin-resistant state and compound the deleterious effects on islet function of higher miR-34a expression. Additionally, the seeming lack of tissue specificity for the effect of rs72631823 on miR-34a expression suggests that results from any future expression quantitative trait loci (eQTL) analysis in an accessible tissue may be justifiably, and with some confidence, extrapolated to more disease relevant, but inaccessible, tissues. The very low MAF and identification exclusively in the Yoruba in Ibadan (YRI) population make eQTL studies for rs72631823, however, a very difficult avenue to pursue.

Given recent studies reporting an association of common genetic variation within pre-miRNAs with type 2 diabetes susceptibility [26] and an enrichment of T2D GWAS signals in genes predicted to be targeted by islet-expressed miRNAs [27], the importance of miRNAs to maintenance of beta-cell function cannot be overstated. Indeed, the results of this study may justify analysing a far larger number of SNPs within miRNA genes (perhaps limited to those expressed in the pancreatic islet) and assessing their effects on miRNA expression. Whilst their rarity impedes the finding of significant associations in single-marker tests, methods such as the one presented here can be used to functionally annotate variants for grouping in collapsed/multiple marker tests, which are more likely to uncover significant associations with complex traits, such as type 2 diabetes.
